# Supplementary material for: Rapid turnover of life-cycle-related genes in the brown algae
Source: Genome Biol. 2019 Feb 14;20:35. doi: 10.1186/s13059-019-1630-6 (PMC6374913; doi:10.1186/s13059-019-1630-6)
Supplement: Supplementary file 3 — Protein alignments, corresponding Gblocks html file and CDS sequences for the lineage conserved gametophyte-biased genes. (ZIP 2109 kb) [file 13059_2019_1630_MOESM3_ESM.zip › Con_GA_alignments/Ec-06_009760.aa_ali.fasta-gb.htm]

Ec-06\_009760.aa.fasta.trans.aa\_ali.fasta


## Gblocks 0.91b Results

Processed file: **Ec-06\_009760.aa.fasta.trans.aa\_ali.fasta**  
Number of sequences: **2**  
Alignment assumed to be: **Protein**  
New number of positions: **667** (selected positions are underlined in blue)

```
                         10        20        30        40        50        60
                 =========+=========+=========+=========+=========+=========+
Ec-06_009760     --------------------------------------------------------MPSV
MSTRG.13146.1    LSYASWAKDAKLRERCKGQCEVMSAKASAGSPVAAEFGYRHVVLFLFVLVLYIYTAMPSV
                                                                         ####


                         70        80        90       100       110       120
                 =========+=========+=========+=========+=========+=========+
Ec-06_009760     PGGDAGELLASGCQLGTPHPP---------------------------------------
MSTRG.13146.1    PGGDAGELLAEGCHLGSPHPPGYPLLNIIYFSLSRLPLPSMLPPTVCADSTPATVSWAAN
                 #####################                                       


                        130       140       150       160       170       180
                 =========+=========+=========+=========+=========+=========+
Ec-06_009760     ---------AAVLVHLTIEEWTHSSVQAYNSGAASAAALLFSFSPLAWEYNTGSEVFALN
MSTRG.13146.1    MLSGGFGSVTAVLASLTVEEWTRASPEAYNVGAAAAASLMFSLSPLAWEYNTGSEVFSLN
                           ##################################################


                        190       200       210       220       230       240
                 =========+=========+=========+=========+=========+=========+
Ec-06_009760     NVLVAVALYVTARIVTRPSMTSARIGAV-------------------------ALTEESL
MSTRG.13146.1    NVLVAASAYLTVRIVMKPNMNLARIGAVVCGLALSNQHSACLVLGPLAICVLRALAEESL
                 ############################                         #######


                        250       260       270       280       290       300
                 =========+=========+=========+=========+=========+=========+
Ec-06_009760     LSCRSLAELGGLWFPLGLSPHAALVVSSRNPQQGSWGDLSSTAGFLRHVLRSEYGTLNLG
MSTRG.13146.1    LSYRTLAELGGLWFPVGLSPYATLLLSARRPQQGSWGDVSSVQGFLRHVLRAEYGTLKLG
                 ############################################################


                        310       320       330       340       350       360
                 =========+=========+=========+=========+=========+=========+
Ec-06_009760     IPVPNAEGAVERIGEYFMDSSSQTMHVGPPMAALGVGWALVVAHTGAHSDKHRQHSRRVR
MSTRG.13146.1    IPDSNSEGALERILEYLVDSSRQTMHLGLPMAVLGIGWAMTSRHEVVTSTKQRQEIRRVR
                 ############################################################


                        370       380       390       400       410       420
                 =========+=========+=========+=========+=========+=========+
Ec-06_009760     HFGLGLATAWAFYVTIWHCIFSNISLHRPMSRAVHARFWIQPNLLLCVAAGCGLGLAANT
MSTRG.13146.1    AFSVGMTAAWAFYVAIWHSVFSNISLHHPMSRAVHGRFWIQPNLLLCLVAGGGLGLVANT
                 ############################################################


                        430       440       450       460       470       480
                 =========+=========+=========+=========+=========+=========+
Ec-06_009760     IAAKLWSVCPSSCRLGKRFAGASVSLLMPTVISTTMFWQRWDSMDRGSWIGGSHGWTMHL
MSTRG.13146.1    IASFLWRGLRR-CRLSKDAVTAGVSLLLPSMLTAAILWLRWDMLDSGAWGGRSHGWTVHL
                 ######      ################################################


                        490       500       510       520       530       540
                 =========+=========+=========+=========+=========+=========+
Ec-06_009760     YGQALLSALPQRALLLSHTDLDLNAARYLRVCEGLRTDVTHISLQMMPYPWWESTQADLH
MSTRG.13146.1    YGQALLSALPEGALLLSHTDLDLNPARYLRVCENLRTDVTHISIQMMPYPWWDSVQAELH
                 ############################################################


                        550       560       570       580       590       600
                 =========+=========+=========+=========+=========+=========+
Ec-06_009760     PGVVFPPIFRGVSTRRESEGNAALVTRFLAANLPQGRQIFLDMQAVDEREIGAVGFYRGF
MSTRG.13146.1    AGVIFPPILRGVNTRRNSEGNAVLITRFLAANLQRGRPIFLDMQAVDESEIGSAGLYRGF
                 ############################################################


                        610       620       630       640       650       660
                 =========+=========+=========+=========+=========+=========+
Ec-06_009760     SLVPHGLVYRVLPRLTLQESESWHEGVVKQLRSLSECMQPVAVSRYRPGTWEFSAASVYW
MSTRG.13146.1    SLVPHGLTYLVLPKLTLQESERWHEEIVKQLRSLDECMEPVSANRYRPGTWEFAAASVYW
                 ############################################################


                        670       680       690       700       710       720
                 =========+=========+=========+=========+=========+=========+
Ec-06_009760     DAHYQAGLFFLSYAIGAWDNAPRATQRPHLLPALRDASALLQRTLEAADEHGTFSSSRSD
MSTRG.13146.1    DAHYQAGLFFLSYGIGVWESAPRAEQRPHLLPALRDASALLLRTLEAVDAHGTFSSSRPD
                 ############################################################


                        730       740       750       760       770       780
                 =========+=========+=========+=========+=========+=========+
Ec-06_009760     LLKNTVLAKIRLLQALQAEAASNP--LSKKHSSSVDSVSHTERRYAEIILAKEHCLTLIP
MSTRG.13146.1    LLKNTILAEIRYLQALQAEAVGMPHLRSKSEPSSVEMASRMEGLFAEMVKAKERCVRLIP
                 ########################   #################################


                        790       800       810
                 =========+=========+=========+
Ec-06_009760     RFLLEAIDDKDAEAFGKVYVKLRETSHM--
MSTRG.13146.1    RFMKGAINDKDAEAFGKVYVKLRGESYQWG
                 ##########################
```

```
Parameters used
Minimum Number Of Sequences For A Conserved Position: 2
Minimum Number Of Sequences For A Flanking Position: 2
Maximum Number Of Contiguous Nonconserved Positions: 8
Minimum Length Of A Block: 10
Allowed Gap Positions: None
Use Similarity Matrices: Yes
```

```
Flank positions of the 5 selected block(s)
Flanks: [57  81]  [131  208]  [234  426]  [433  744]  [748  806]  

New number of positions in Ec-06_009760.aa.fasta.trans.aa_ali.fasta-gb:  667  (82% of the original 810 positions)
```
